# The Facile Strategy of Improving the Long-Term Stability of Highly Transparent Polyvinyl Chloride by Introducing Unsaturated Zn Oleate and Uracil Derivatives

**DOI:** 10.3390/ma15072672

**Published:** 2022-04-05

**Authors:** Lifang Song, Huiwen Huo, Wenshuo Zhang, Huiyun Xia, Yanhui Niu

**Affiliations:** Engineering Research Center of Transportation Materials of Ministry of Education, School of Materials Science and Engineering, Chang’an University, Xi’an 710064, China; huohuiwen@outlook.com (H.H.); z15534946700@163.com (W.Z.); xiahy@chd.edu.cn (H.X.)

**Keywords:** polyvinyl chloride, highly transparency, zinc oleate, uracil derivatives, thermal stability

## Abstract

In order to improve the initial color and the long-term heat stability of super-transparent polyvinyl chloride (PVC), a series of composite heat stabilizers consisting of unsaturated Zn oleate and uracil derivatives have been designed in this paper. The uracil derivatives are 1,3-dimethyl-6-amino-uracil (DAU) and 6,6′-diamino-1,1′,3,3′-tetramethyl-5,5′-(ethylidene)bisuracil (OSU). The static thermal stability, dynamic thermal stability, and transparency were used to evaluate the properties of the stabilized transparent PVC sheets. The results indicate that the compatibility between the stabilizer and PVC was greatly enhanced by introducing an unsaturated long-chain Zn oleate and a long alkyl chain bisuracil derivative. Through the thermal discoloration test, the best ratio of DAU/zinc oleate (DAU/Zn) and OSU/zinc oleate (OSU/Zn) was determined to be 4:1, with a total amount of 3 phr in 100 phr PVC. It was verified that the combination of zinc oleate with uracil derivatives could improve the long-term thermal stability of PVC, and the DAU/Zn was better than that of the OSU/Zn. In addition, through the transmission/haze verification, adding a proper amount of epoxidized soybean oil (ESBO) and phosphite ester to the OSU/Zn system has a certain synergistic effect. The thermal stability and transparency of PVC can be remarkably enhanced.

## 1. Introduction

Polyvinyl chloride (PVC), a kind of widely used thermoplastic polymer, is polymerized from vinyl chloride monomer through the mechanism of free radical chain polymerization. PVC-related products have attracted wide attention due to their low prices, good mechanical properties, and durable physical and chemical properties such as chemical resistance, abrasion resistance, and non-combustibility [1,2,3]. The processing temperature of PVC is often over 170 °C, while its decomposition temperature is about 140 °C, so PVC is easily degraded when heated or sheared during processing [4,5,6]. Therefore, a certain amount of heat stabilizer should be considered during the molding process to ensure the performance of PVC [7].

Among the commercial PVC heat stabilizers, heavy metal salt heat stabilizers are cheap and have better stabilizing effects, but heavy metals cause serious environmental pollution and can quickly enter the human body through various channels and cause harm, which does not meet the requirements of product quality standards. Organotin heat stabilizers are expensive, and the processing process will produce toxic substances of varying degrees. Rare earth heat stabilizers have poor early stability and are easily precipitated [8,9,10]. Calcium–zinc heat stabilizers are recognized as environmentally friendly heat stabilizers, but due to the effect of “zinc burning,” there are certain defects in long-term stability [11]. However, organic stabilizers are relatively novel, low-toxic, highly effective, and have good development prospects. Therefore, researchers have gradually turned their attention to organic stabilizers. Most of the PVC stabilizers used at present are composite systems designed and developed based on the principle of complementary synergy. Among them, calcium–zinc stabilizers and organic heat stabilizers exhibit synergistic effects [12,13,14].

Uracil and its derivatives are closely related to the human life cycle, ensuring the environmental protection of environmentally friendly heat stabilizers [15,16,17]. At present, nitrogen-containing heterocyclic heat stabilizers are organic heat stabilizers that have been studied more. These heat stabilizers have a great possibility to be developed into main stabilizers. Uracil and its derivatives have been investigated more and more in PVC heat stabilizers [18,19,20]. Mohamed and Sabaa studied barbituric acid and its derivatives as nitrogen-containing heterocyclic heat stabilizers individually. Compared with the commercially available lead salt and organotin, they found that when the heat stabilizer was added, the HCl release rate of PVC was significantly reduced [21,22]. Mohamed et al. used benzimidazole and anthrone as heat stabilizers to prepare PVC. However, the stabilizing effect was mediocre, and the prepared derivatives could not be used as the main stabilizer [23,24,25,26]. Xu et al. used 3-amino 1,2,4-triazole as a PVC heat stabilizer. Although it does not have excellent heat stability, this type of heat stabilizer has sterilization properties, which dramatically expands the use of PVC [27]. Wu et al. tried to introduce long aliphatic chains on 6-amino-1,3-dimethyluremiidine, which increased the compatibility between the stabilizer and PVC. The stabilizer was able to disperse uniformly in PVC and the efficiency of heat stabilization was improved. The light transmittance and haze of the PVC samples were greatly enhanced, which provides a new strategy for the design of heat stabilizers for transparent PVC. However, the long-term stability needs to be further improved [28,29]. As a matter of fact, most industrialized PVC stabilizers are composite systems based on the principle of complementary synergy.

Zinc oleate is an unsaturated acid salt compound. The double bond in the chains can undergo a diene addition reaction with the conjugated double bond in the PVC macromolecule, which can prevent the continued growth of the conjugated polyene segment and reduce the initial coloration of PVC. Both uracil and its derivatives have been reported to exhibit good heat stabilities to PVC. The nitrogen in the chains can replace the unstable chlorine atoms in the PVC molecule and absorb the initial decomposition product, hydrogen chloride. Among them, zinc oleate is a heat stabilizer that is beneficial for alleviating the initial whiteness of PVC, and uracil is a heat stabilizer with long-term thermal stability. By combining the two heat stabilizers, it is possible to achieve both the initial whiteness and long-term stability. In addition, uracil derivatives and zinc oleate are formulated with other heat-stabilizing additives to achieve high transparency.

**Scheme 1:**
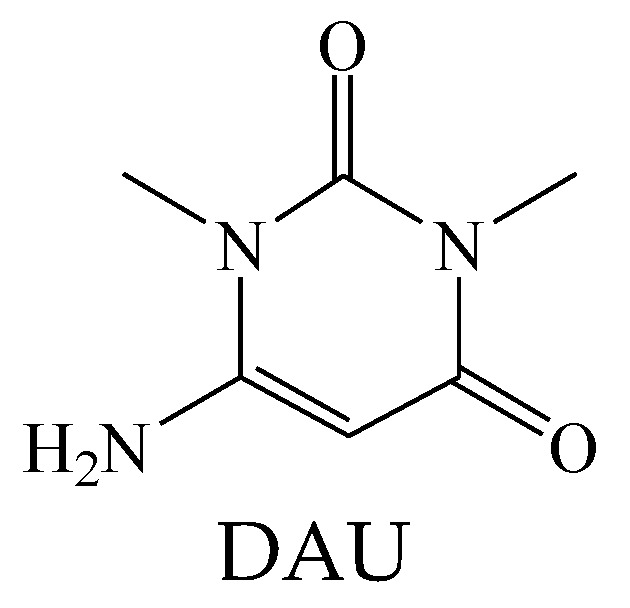

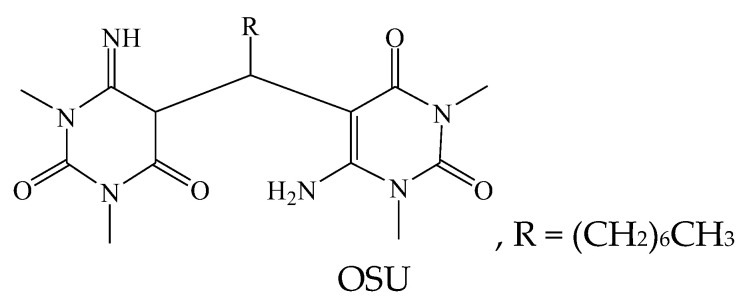
The structures of DAU and OSU.

In this paper, cheap and readily available zinc oleate and uracil derivatives are used as raw materials as the main thermal stabilizer of PVC. A one-pot reaction is used to prepare the zinc oleate heat stabilizer. The uracil derivatives are 1,3-dimethyl-6-amino-uracil (DAU) and 6,6′-diamino-1,1′,3,3′-tetramethyl-5,5′-(ethylidene)bisuracil(OSU), the structures of these two compounds are as shown in Scheme 1. Thus, the thermal stability and transparency of DAU/Zn- and OSU/Zn-stabilized PVC were investigated, and then the addition of ESBO and phosphite ester was discussed. The composite heat stabilizer, ESBO, and phosphite ester possessed a good synergistic effect.

## 2. Experimental

### 2.1. Materials

Polyvinyl chloride (SG-5) was purchased from Zhengbang Chemical Co., Ltd., (Wenzhou, China). Zinc oxide was provided by Sinopharm Chemical Reagent Co., Ltd., (Shanghai, China). Plasticizer (DINCH, A.R.) was purchased from Kaiyin Chemical Co., Ltd., (Shanghai, China). Phosphite ester was purchased from Shandong Yousuo Chemical Co., Ltd., (Linyi, China). No. 25 transformer oil was purchased from Mojiezuo Petrochemical Co., Ltd., (Shanghai, China). Octanal (97%), 1,3-dimethyl-6-amino-uracil(DAU) (A.R.), oleic acid (A.R.), diethylene glycol butyl ether (A.R.), epoxidized soya bean oil (C.P.), and the other chemical reagents used in this study were obtained from Aladdin Reagent, (Shanghai, China).

### 2.2. Preparation of Zinc Oleate

Firstly, 2.14 g of oleic acid (0.22 mol) and 24.00 g of No. 25 transformer oil as a solvent were added into a three-necked round-bottom flask, stirred continuously, and heated up to 85 °C. Secondly, 2.00 g of diethylene glycol butyl ether and 8.14 g of zinc oxide were added and reacted continuously at 85 °C for 2.5 h. Finally, the product zinc oleate was distilled under reduced pressure to remove water.

### 2.3. Preparation of Uracil Derivative (OSU)

At first, 1.55 g (10 mmol) of 1,3-dimethyl-6-amino-uracil was stirred and dissolved in suitable deionized water in a 500 mL round-bottom flask. Then, 0.64 g (5 mmol) octanal was added to the above solution and stirred at room temperature for 48 h. Finally, the 6,6′-diamino-1,1′,3,3′-tetramethyl-5,5′-(ethylidene)bisuracil (OSU) was washed with distilled water and dried at 50 °C overnight.

### 2.4. Preparation of PVC Sheets

According to the material formula, PVC powder, composite heat stabilizer, and auxiliary stabilizer by mass were weighed and put into a Chinese herbal medicine grinder (FW135, Test Instrument Co., Ltd., Tianjin, China). The PVC powder with stabilizer was mixed at room temperature for 2 min. The PVC sheets were prepared using a double-roll mill (XL-KLYP1, Xilong Electrical Machinery Equipment Co., Ltd., (Dongguan, China)) at 180 °C.

### 2.5. Characterization

FTIR spectra were performed at room temperature on a Nicolet iS10 FTIR spectrometer (ThermoFisher Scientific, Waltham, MA, USA) using the KBr tablet method. ^1^H NMR spectra were recorded on an AVANCE III 500 MHz spectrometer (Bruker Corporation, Billerica, MA, USA) using DMSO-d6 as a solvent and tetramethylsilane as the internal standard.

### 2.6. Evaluation Methods

#### 2.6.1. Static Thermal Stability

The Congo red test and the discoloration test were used to evaluate the static thermal stability of the PVC sheets.

The Congo red test was carried out in an oil-bath-heated glass tube. An amount of 5.00 g of PVC, 0.15 g of heat stabilizer, and a certain amount of heat-assisted stabilizer were added to a thin glass tube, with the Congo red paper about 2.5 cm over the top of the PVC. The glass tubes were plugged with a stopper and immersed into an oil bath at 180 °C in air. The timing was stopped when the test paper turned blue. The static thermal stability was evaluated based on time.

The discoloration test was conducted in a thermal aging oven. The PVC sheets were cut into samples of about 20 mm × 40 mm, then heated in an aging test oven (BHO-401A, Yiheng Scientific Instruments Co., Ltd., Shanghai, China) at 180 °C in air. The test period was determined by observing the color every ten minutes until it darkened.

#### 2.6.2. Dynamic Thermal Stability

The dynamic thermal stability was tested by the torque rheometer (RTOA-20/10, Putong Experimental Analytical Instrument Co., Ltd., Guangzhou, China). An amount of 60 g of PVC was added to the sample cell, where the rotation speed was 50 r/min, and the sample was tested at 180 °C. The torque changes with time were recorded.

#### 2.6.3. Thermogravimetric Analysis

The thermal stability of the PVC sheets was executed on a thermogravimetric analyzer (Q600, TA Instruments-Waters, USA), heated from room temperature to 600 °C at a rate of 10 °C/min under an air atmosphere.

#### 2.6.4. Transparency

The transparent performance of the PVC sheets was evaluated by the light transmittance, which was measured on the light transmittance/haze meter (HunterLab UltraScan PRO, Shanghai, China).

## 3. Results and Discussion

### 3.1. Characterization of Stabilizers

**Figure 1:**
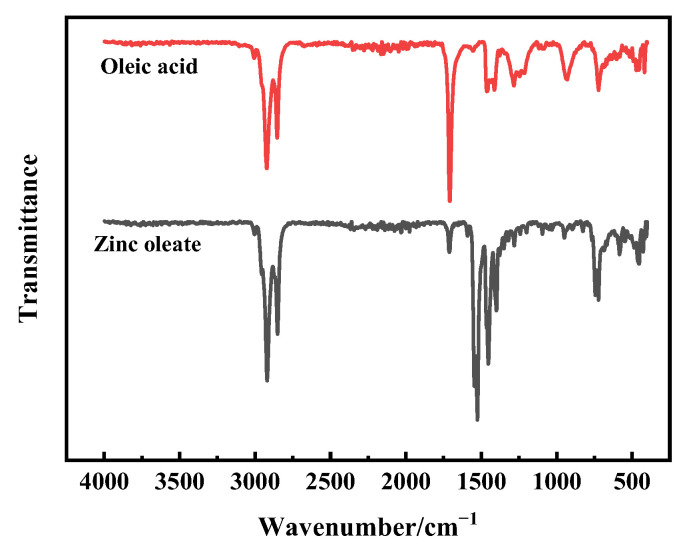
FTIR spectra of the prepared zinc oleate and oleic acid.

The FTIR spectra of zinc oleate and oleic acid are shown in Figure 1. It can be seen that there are two strong absorption peaks near 2800 cm^−1^ and 2900 cm^−1^, which can be assigned to C-H stretching vibration. The -COO- in the carboxylate is a multi-electron conjugated system. Because of the two C = O vibration couplings, there should be no absorption. While the strong absorption peaks appear in two bands, the one at 1560–1610 cm^−1^ is the peak of the antisymmetric stretching vibration, and the other at 1360–1440 cm^−1^ is the peak of the symmetric stretching vibration. The intensities of these two bands are weaker than that of the antisymmetric stretching vibration, usually two or three broad bands, so it can be proved that the compound is zinc oleate.

The absorption peak at about 1700 cm^−1^ in Figure 1 should be unreacted oleic acid. As it is known that oleic acid is a weak acid, the reaction of oleic acid with ZnO is incomplete. Therefore, a little more oleic acid and a longer reaction time are required for a high conversion of ZnO. Besides, appropriate increases of the oleic acid during the synthesis of zinc oleate can also prevent zinc oleate from decomposing.

**Figure 2:**
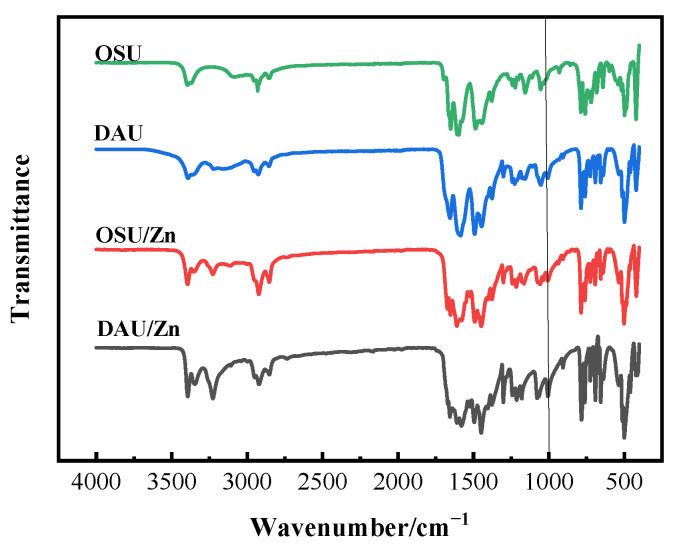
The FTIR spectra of the prepared stabilizers.

Figure 2 shows the infrared spectra of DAU, OSU, and composite heat stabilizers, respectively. The absorption peaks of OSU and DAU are similar and nearly all the peaks are overlapped. The height at 2920 cm^−1^ of OSU is more potent than that of DAU, which belongs to the -CH_2_- stretching vibration. In addition, the ridge at 1000 cm^−1^ of OSU disappears compared to DAU, because of the disappearance of the -CH- trying vibration on the pyrimidine ring, which indicates the successful synthesis of OSU. By comparing the infrared spectra of the composite system, there is no chemical reaction between the composite heat stabilizers before adding to PVC.

**Figure 3:**
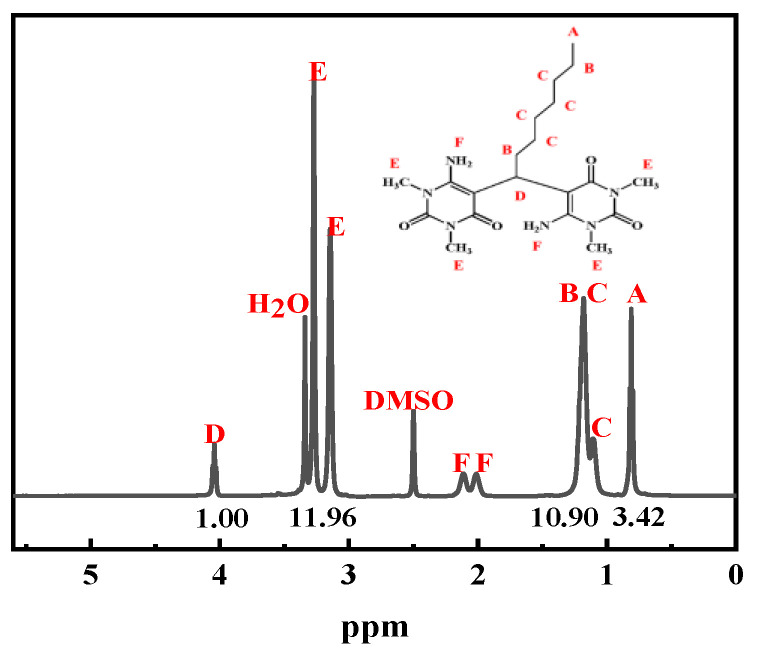
^1^H NMR spectrum of OSU.

The ^1^H NMR spectrum of OSU is shown in Figure 3**.** OSU is a yellowish solid with a yield of 89.3%. ^1^H NMR (500 MHz, DMSO-d6): d = 0.81–0.87 (m, 3H, -CH_3_), 1.08–1.28 (m, 12H, -CH_2_), 2.12(br.s, 4H, -NH_2_), 3.16–3.32 (s, 12H, -NCH_3_), 4.02–4.08 (m, 1H, -CH-). According to the 1H NMR results, 6,6′-diamino-1,1′,3,3′-tetramethyl-5,5-(ethylidene)bisuracil (OSU) was successfully synthesized from 1,3-dimethyl-6-amino-uracil and octanal.

### 3.2. Thermogravimetric Analysis of DAU/Zn- and OSU/Zn-Stabilized PVC

**Figure 4:**
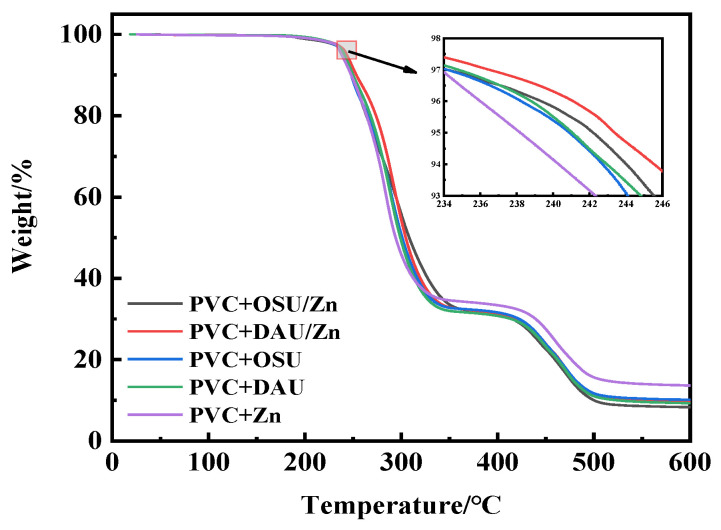
TGA curves of different heat stabilizers stabilizing PVC in air.

Thermogravimetric analyses are used to illustrate the contribution of the main components constituting the composite stabilizer to the thermal stability of PVC. The effects of OSU and DAU as the main stabilizers compounded with zinc oleate on the initial thermal decomposition temperature of polyvinyl chloride sheets are shown in Figure 4. There are 3 phr of additives per 100 phr of PVC.

The TG curves show two steps of weight loss. The first step is due to the dechlorination of the zipper during PVC heating, with a temperature between 230 and 250 °C. Following the dehydrochlorination, a conjugated diene structure is formed, which is considered to be the chromogenic group. The second step is the continuous decomposition of the diene sequence to generate low molecular weight hydrocarbons [30].

It can be seen that the initial decomposition temperature of PVC stabilized with DAU/Zn is greater than that of OSU/Zn, and the decomposition temperatures of these two compound systems are higher than a single heat stabilizer compound, indicating that the two compound systems stabilizing PVC have higher thermal degradation stability. In addition, we can also find that the curves of the DAU/Zn and OSU/Zn systems are similar, which shows that the thermal stability of PVC did not essentially reduce when the long-chain alkanes were introduced into the heat stabilizer.

### 3.3. Influence of Main Stabilizer Composition on Heat Stabilizer Performance

**Figure 5:**
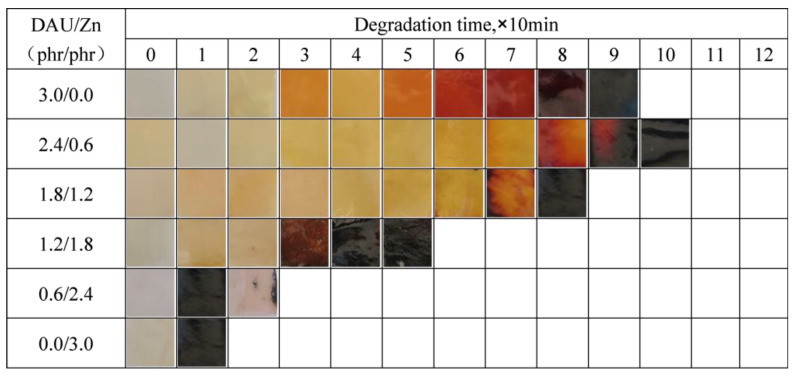
Discoloration of PVC sheets stabilized by different ratios of DAU/Zn at 180 °C.

**Figure 6:**
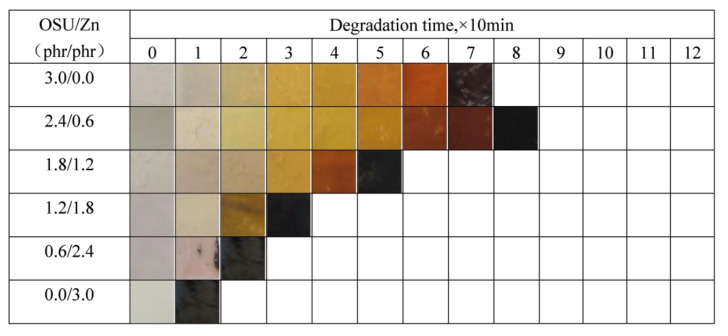
Discoloration of PVC sheets stabilized by different ratios of OSU/Zn at 180 °C.

Figure 5 and Figure 6 show the discoloration of PVC sheets stabilized by different compositions of DAU/Zn and OSU/Zn. The proportion of the main stabilizers, OSU/Zn, DAU/Zn, OSU, and DAU is 3 phr per 100 phr of PVC.

It can be seen that when 3 phr of zinc oleate is used as a single heat stabilizer, the initial whiteness of the PVC sample is the best, but it turns black completely after being heated at 180 °C for just 10 min. Zinc oleate is a typical initial heat stabilizer for PVC processing. It can effectively replace the unstable chlorine atoms on the PVC chain with carboxylates, which inhibit the initial degradation and coloring of PVC sheets. In addition, the degradation product of zinc oleate is ZnCl_2_, an efficient catalyst for accelerating the thermal decomposition of PVC, which causes malignant degradation and makes it suddenly turn black, resulting in poor long-term stability [31].

When 3 phr of DAU and OSU were used as single stabilizers, respectively, the comparison revealed that the initial whiteness of the samples of PVC with OSU’s addition was better because the long-chain alkyl groups introduced in OSU were more compatible with PVC. The initial whiteness of both systems was improved after compounding because of the favorable initial whiteness of zinc oleate. Comparing DAU/Zn with OSU/Zn, the initial whiteness is still better for OSU/Zn.

Long-term static thermal stability reflects a more realistic process and is therefore important for PVC processing. It can be seen from Figure 5 that with the increase in the DAU content in the composite stabilizer DAU/Zn, the long-term stability of the PVC sample gradually increases, and the initial whiteness does not decrease significantly. When the ratio of DAU/Zn reaches 2.4/0.6, the stabilizing effect of the composite stabilizer is the best, up to 100 min. On the one hand, DAU can not only absorb HCl but also replace the unstable chlorine atoms. Furthermore, DAU can react with zinc oleate to form a metal complex during PVC processing, which makes ZnCl_2_ lose its catalytic activity and dramatically enhances the long-term stability of PVC samples.

It can be seen from Figure 6 that as the ratio of OSU/Zn in the stabilizer increases, the long-term stability of the PVC sample increases gradually. This may be attributed to the synergy of OSU/Zn. The initial whiteness performs better, which may be because of the reaction of OSU and zinc chloride. Meanwhile, the long-term stability of OSU/Zn is not as good as DAU/Zn, which may be due to how the introduction of long-chain alkanes in uracil has little contribution to the stability of PVC. The amino-functional groups of uracil and bisuracil exert a stabilizing effect on PVC.

### 3.4. Mechanism of the Main Stabilizer to Stabilize PVC

FTIR was carried out to verify whether the OSU replaces the unstable chlorine in PVC. The PVC sheet was dissolved in a tetrahydrofuran solution and filtrated to remove the unreacted OSU. The purified PVC sample for the FTIR was precipitated through methanol from the above filtrate and heated at 180 °C for different lengths of time.

**Figure 7:**
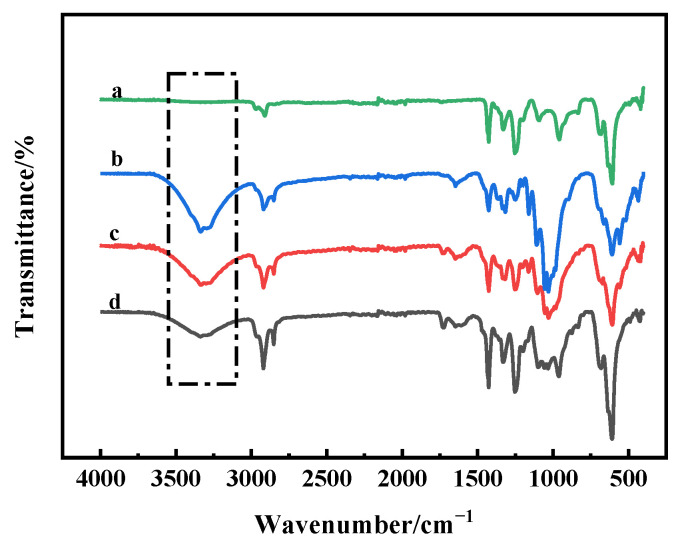
The FTIR spectra of pure PVC (**a**) and purified PVC after aging at 180 °C for (**b**) 10 min, (**c**) 30 min, (**d**) 50 min.

**Scheme 2:**
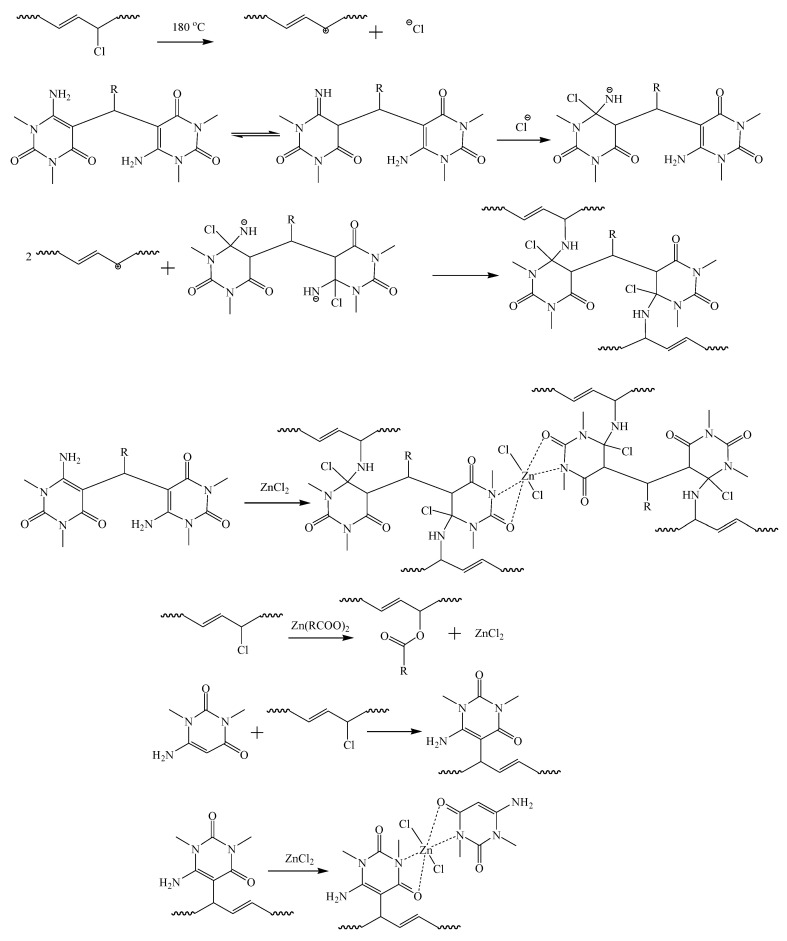
A probable mechanism of the stabilization action of OSU/Zn and DAU/Zn.

Figure 7 shows the FTIR spectra of pure PVC and purified PVC aging at 180 °C for different lengths of time (10 min, 20 min, and 30 min). In the spectrum of the purified PVC after aging at 180 °C for 10 min (Figure 7b), absorption peaks can be found between 3400 cm^−^^1^ and 3200 cm^−^^1^, contributed by amino groups. With the increase in aging time, the absorption peak of the amino group gradually weakens, which may be because the amino group continuously replaces the unstable chlorine. This indicates that the amino group in the OSU reacted with the unstable chlorine in the PVC. The mechanism of the main stabilizer to stabilize PVC is as shown in Scheme 2.

### 3.5. The Effect of ESBO and DINCH on PVC Properties

The thermal stability of the PVC sheets was further investigated by choosing auxiliary stabilizers, ESBO and DINCH. ESBO is a widely used PVC non-toxic plasticizer and stabilizer, which has a good synergistic effect [29]. DINCH is an environmentally friendly plasticizer widely used in PVC processing.

The addition of OSU/Zn as the main stabilizer was 3 phr, while the combined amount of ESBO and DINCH was 30 phr and kept unchanged. The addition of ESBO was gradually increased to 0, 3, 6, and 9 phr, and the corresponding DINCH proportions were 30, 27, 24, and 21 phr, respectively, as shown in Table 1.

#### 3.5.1. Static Thermal Stability of PVC with Different Amounts of ESBO

**Figure 8:**
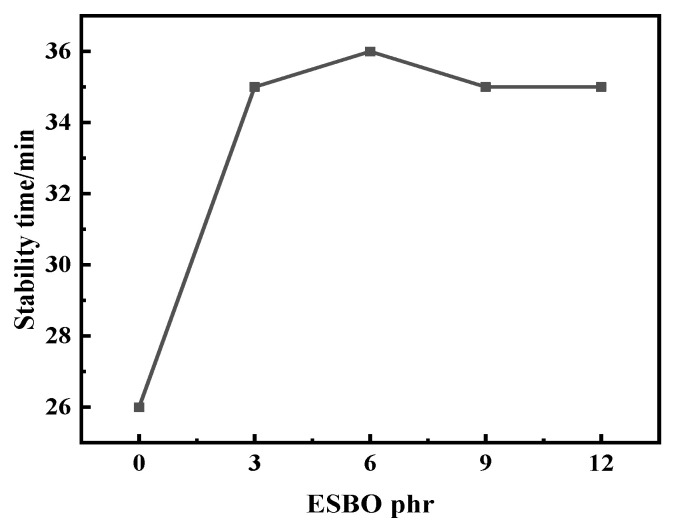
Thermal stabilization time of PVC samples stabilized with thermal stabilizers containing different proportions of ESBO at 180 °C.

The effects of the amounts of ESBO on the static thermal stability of PVC are evaluated by the Congo red test. Figure 8 shows that when ESBO is not added, the test time is 26 min. With the increase in the ESBO proportion, the PVC stabilization time increases continuously. When 6 phr of ESBO was added, the thermal stability was the best. It can be found that adding too much ESBO cannot continuously improve the strength of PVC, indicating that a specific part of ESBO has benefits for preventing PVC degradation.

#### 3.5.2. Dynamic Thermal Stability of PVC with Different Amounts of ESBO

**Figure 9:**
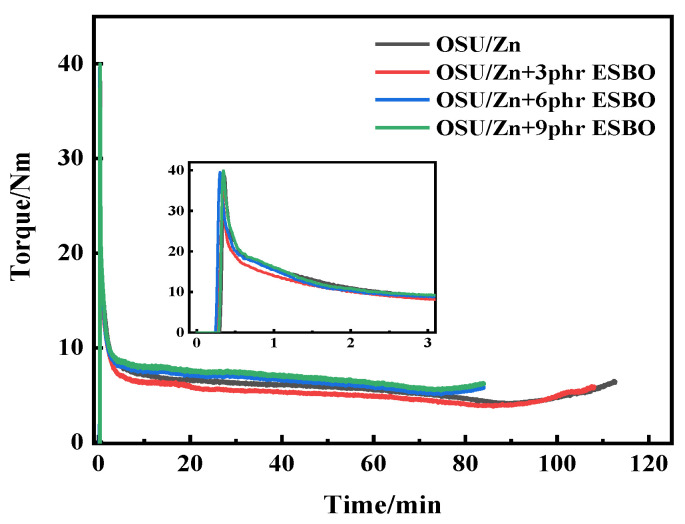
Dynamic thermal stability of PVC with different amounts of ESBO addition, the rotor speed is 50 rpm.

The dynamic thermal stability of PVC measured by torque rheometer is a way to reflect the rheological properties of the polymer in actual processing [32]. Figure 9 shows the dynamic thermal stability of PVC with different amounts of ESBO. The thermal degradation time of the PVC sample did not change much when ESBO was added at 3 phr. When the amount of ESBO continued to increase, the degradation time of the PVC sample was significantly shortened. This may be because the plasticizing ability of ESBO is not as good as DINCH, which leads to an increase in shear heat in the mixing chamber of the torque rheometer [33].

We found that ESBO has some kind of auxiliary heat stabilizer effect. When the ratio of ESBO and DINCH is 3:27, the static heat stability of PVC reaches 35 min, and the dynamic heat stability reaches about 90 min.

### 3.6. The Effect of Phosphite Ester on the Performance of PVC

To further improve the thermal stability of PVC sheets, phosphite ester was introduced to stabilize the PVC sheets. Phosphite ester is often used to stabilize transparent PVC, but the thermal stability is poor if the main stabilizer is not added, which is similar to the single usage of ESBO. Phosphite ester can not only absorb HCl but also can chelate metal ions or react with metal chlorides to generate phosphite when used in combination with metal stabilizers in PVC heat stabilizers, reducing its catalytic degradation effect on PVC degradation. In addition, the additional reaction between phosphate and the conjugated diene structure in PVC can alleviate the degradation and coloring.

**Figure 10:**
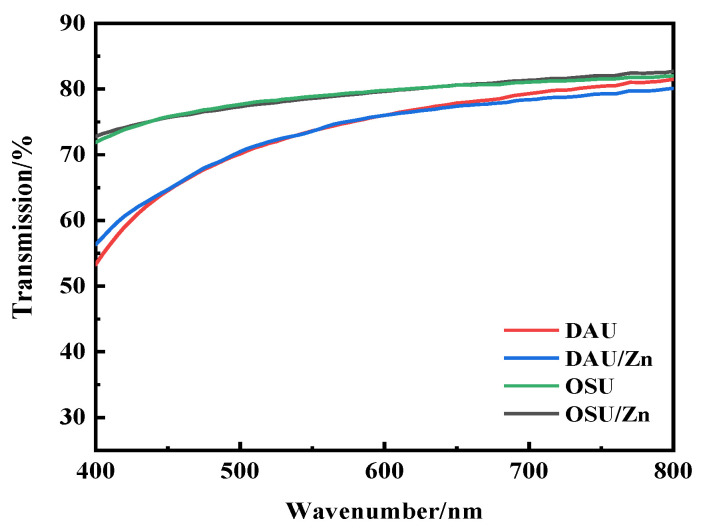
Visible light transmittance of PVC sheets stabilized with different main stabilizers.

Transparency in the application of PVC products is an essential factor that should be considered [34]. As can be seen from Figure 10, the visible light transmittance of OSU is significantly higher than that of DAU. The long aliphatic chains of OSU increase the compatibility of the stabilizer with PVC and effectively reduce their impact on the transparency of the PVC sheets [13,35]. The visible light transmittance of the composite system was further compared with that of the single compound, indicating that adding zinc oleate into the hybrid system did not reduce the visible light transmittance of the system.

Whether the addition of phosphite ester has an effect on the thermal stability and transparency of PVC was carried out on the basis of 3 phr of the main stabilizer as well as 3 phr of ESBO and 27 phr of DINCH.

**Figure 11:**
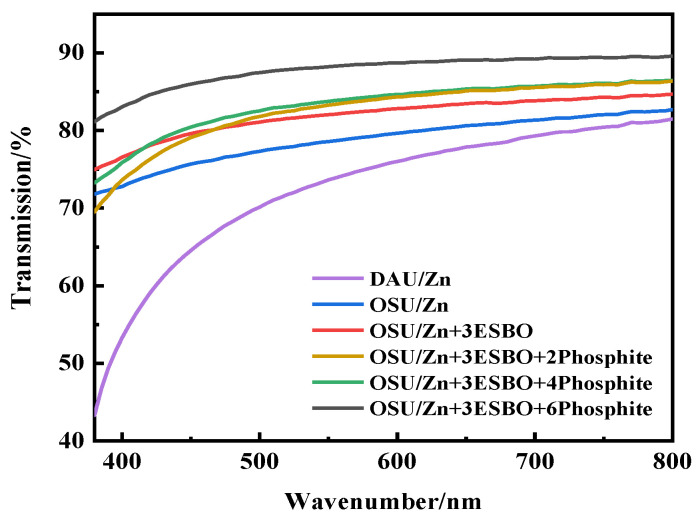
Visible light transmittance of PVC sheets stabilized with different composite stabilizers.

**Figure 12:**
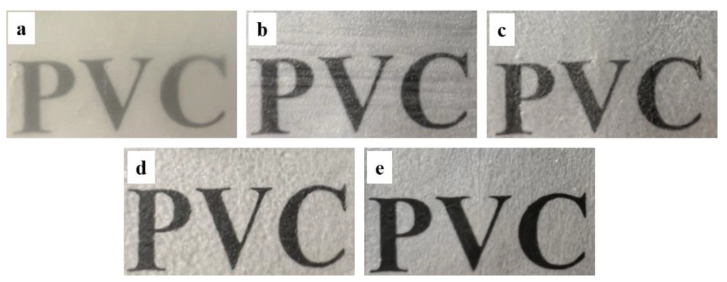
The optical photos of PVC sheets’ transparency stabilized by (**a**) 30 phr of DINCH, 3 phr of OSU/Zn; (**b**) 27 phr of DINCH, 3 phr of ESBO, 3 phr of OSU/Zn; (**c**) 27 phr of DINCH, 3 phr of ESBO, 3 phr of OSU/Zn, 2 phr of phosphite ester; (**d**) 27 phr of DINCH, 3 phr of ESBO, 3 phr of OSU/Zn, 4 phr of phosphite ester; (**e**) 27 phr of DINCH, 3 phr of ESBO, 3 phr of OSU/Zn, 6 phr of phosphite ester.

Figure 11 shows that with the continuous increase in the phosphite ester amount, the visible light transmittance of PVC sheets increases continuously. When the phosphite ester was increased to 6 phr, the visible light transmittance of the PVC sheet was the best. In addition, it can be seen from the optical image in Figure 12 that the visible light transmittance of the PVC sheet reaches the highest when there is 6 phr of phosphite ester. The synergistic effect of ESBO and OSU/Zn significantly improves the transparency of PVC sheets. The transparency is greatly improved when ESBO is used together with phosphite, which also indicates a better synergistic effect.

**Figure 13:**
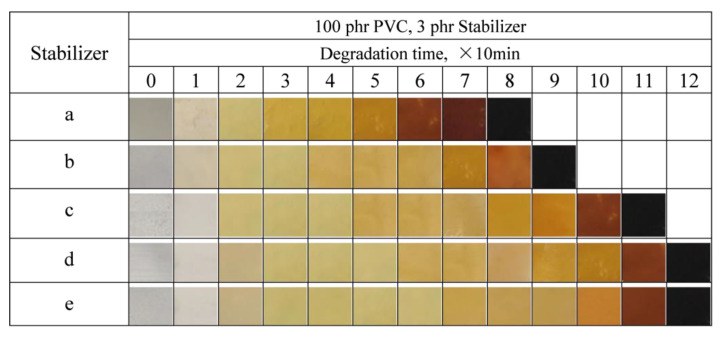
Discoloration of PVC sheets stabilized by (**a**) 30 phr of DINCH, 3 phr of OSU/Zn; (**b**) 27 phr of DINCH, 3 phr of ESBO, 3 phr of OSU/Zn; (**c**) 27 phr of DINCH, 3 phr of ESBO, 3 phr of OSU/Zn, 2 phr of phosphite ester; (**d**) 27 phr of DINCH, 3 phr of ESBO, 3 phr of OSU/Zn, 4 phr of phosphite ester; (**e**) 27 phr of DINCH, 3 phr of ESBO, 3 phr of OSU/Zn, 6 phr of phosphite ester.

In order to investigate whether the addition of ESBO and phosphite ester adversely affects the thermal stability of transparent PVC sheets, a discoloration test of PVC sheets was conducted in a thermal aging oven. Figure 13 shows the discoloration test of PVC after adding ESBO, phosphite ester, and stabilizers at different amounts. The transparency was improved after adding ESBO. With the addition of 3 phr of ESBO, the long-term thermal stability of PVC products increases continuously with the increase in the phosphite ester amount. The performance and initial transparency of PVC are improved. The PVC sheet containing 6 phr of phosphite ester has the best initial color and transparency, probably because of the high synergy of OSU/Zn, ESBO, and phosphite ester, which can effectively inhibit the degradation of PVC.

## 4. Conclusions

In summary, a series of composite heat stabilizers with long-term stability for transparent PVC sheets have been presented. The composite heat stabilizer consists of zinc oleate and uracil or its derivatives. The results show that the composite heat stabilizer shows good initial whiteness and long-term thermal stability. The thermal stability of the DAU/Zn system is better than that of the OSU/Zn system. The visible light transmittance of the OSU/Zn system is better than that of the DAU/Zn system. Adding a proper amount of ESBO to the OSU/Zn system improves the thermal stability and transparency of PVC. The addition of phosphate during the preparation of PVC sheets can provide better optical transparency. During the experiment, it was found that adding three parts of OSU/Zn, three parts of ESBO, and six parts of phosphate can maximize the transparency of the PVC sheet. The composite heat stabilizer has practical application value for processing highly transparent plasticized PVC products in the future.

## Figures and Tables

**Table 1:** Polyvinyl chloride formula.

PVC/g	OSU/Zn/g	Plasticizer/g	
		ESBO	DINCH
100	3	0	30
100	3	3	27
100	3	6	24
100	3	9	21

## Data Availability

Data sharing is not applicable to this article.
